# Functionalized Modified Ti_4_O_7_ Polyaniline Coating for 316SS Bipolar Plate in Proton-Exchange Membrane Fuel Cells

**DOI:** 10.3390/polym16182592

**Published:** 2024-09-13

**Authors:** Ting Zhao, Zibin Chen, Xiaoqi Yi, Enfeng Huang, Yanli Wang

**Affiliations:** Guangxi Key Laboratory of Electrochemical Energy Materials, School of Chemistry and Chemical Engineering, Guangxi University, Nanning 530004, China

**Keywords:** proton-exchange membrane fuel cell, PANI, Ti_4_O_7_, coating, corrosion

## Abstract

In this paper, the PANI/PDA-Ti_4_O_7_ composite coating was prepared on 316L by constant current deposition with a current density of 2.8 mA·cm^−2^, in which the Ti_4_O_7_ powders were modified by PDA (polydopamine). The open-circuit potential of the obtained PANI/PDA-Ti_4_O_7_ composite coating is about 365 mV_Ag/AgCl_, which is more positive than that of the bare 316L. During immersion in 1 M H_2_SO_4_ + 2 ppm HF for 200 h, the high stable corrosion potential and the lower *R*_f_ indicate that the composite coating has long-term corrosion resistance.

## 1. Introduction

The proton-exchange membrane fuel cell (PEMFC) has been widely developed because of its advantages of high energy conversion efficiency, good cleanliness, and low noise [1,2]. It is a device that converts chemical energy directly into electrical energy through electrochemical reactions, making it very suitable for use as a mobile power system. A bipolar plate is one of the key components of PEMFCs, which plays the role of connecting the single battery, conducting the current, and separating the gas into the anodic and cathodic sides [3]. Due to the existence of corrosive ions such as SO_4_^2−^, F^−^, and HCO_3_^−^ in the internal working environment of PEMFCs, the bipolar plate needs to have certain electrical conductivity and corrosion resistance [4]. The commonly used bipolar plate materials mainly include graphite, composite, and metallic material. Among them, metallic materials possess high mechanical strength, good electrical and thermal conductivity, and are easily machined into thin sheets to greatly improve the specific power of the stack, thus becoming a very competitive bipolar plate material [5]. However, metallic materials are prone to corrosion or passivation in a weakly acidic PEMFC working environment, which affects the output power of the battery [6]. Applying a protective coating on its surface is an economical and effective approach. 

Researchers at home and abroad have proposed many types of conductive and corrosion-resistant coatings, which are mainly divided into carbon-based coatings (i.e., graphite, diamond, and conductive polymers) and metal-based coatings (i.e., precious metals, metallic carbides, and metallic nitrides) [7,8]. Among them, conductive polyaniline coating is a type of promising surface-protective coating for metallic bipolar plates due to its simple preparation process, adjustable conductivity, and good compatibility with the carbon gas diffusion layer [9,10,11]. Conductive polyaniline (PANI) has become a research hotspot in the field of electrode materials in recent years because of its good oxidation/reduction characteristics, high charge storage ability, and no dendrites [12,13,14]. In addition to acting as a physical barrier layer on the metal surface, polyaniline can also passivate the metal matrix to further improve the protective ability. However, the polymer itself has high porosity; thus, the coating cannot effectively block the penetration of corrosive ions during long-term service, which accelerates the corrosion rate of the matrix [15]. In addition, the de-doping process also occurs for the conductive polymer itself in order to balance the charge, thus reducing the conductivity [16]. The simultaneous reduction in corrosion resistance and electrical conductivity is a fatal disadvantage for the protective coating on the bipolar plate surface. Therefore, a polyaniline/inorganic particle composite coating is considered to be an effective method to reduce the porosity and improve the corrosion resistance. Jafari et al. prepared a graphene-nanoparticle-modified polyaniline coating by cyclic voltammetry on a copper electrode [17]. The evenly distributed graphene nanoparticles decreased the porosity of the polyaniline coating; thus, the inhibition efficiency was increased to 98%. Carbon tubes were also used to improve the corrosion resistance and electrical conductivity through reducing the corrosion current density by an order of magnitude and the interfacial contact resistance by 35% [14]. In addition, TiO_2_ oxide particles were also commonly used to modify the polyaniline coating. PANI/TiO_2_ nanocomposite coatings were prepared by in situ polymerization and it was found that the corrosion potential of the 4.5 µm PANI/TiO_2_ coating increased to 0.080 V and the porosity was only 0.07%. However, TiO_2_ was a semiconductor, which could limit the effective delocalization of the charge, thus decreasing the conductivity of the composite [18,19]. 

As a titanium oxide with a Magneli phase, Ti_4_O_7_ is characterized by high electrical conductivity (1995 S·cm^−1^) and high chemical stability in acid solutions, making it an ideal modification material for a polyaniline coating on bipolar plates. Zhu [20] found that the addition of Ti_4_O_7_ effectively improved the catalytic activity and stability of a Pd-WC-Ti_4_O_7_/C composite electrode. Gao [21] added Ti_4_O_7_ to lead powders to make a modified positive electrode and found that the addition of Ti_4_O_7_ not only increased the oxygen evolution overpotential but also increased the battery capacity and cycle times. However, if the Ti_4_O_7_ powders are simply physically mixed with the polyaniline coating, the effect is limited due to the problem of particle dispersion. 

Dopamine (DA) is a green and environmentally friendly organic reducing agent that can be polymerized to form a polydopamine (PDA) layer with good adhesion and can form a stable PDA coating on the surface of most materials [22]. The molecular structure of DA contains abundant amino and catechol groups, which can provide active modification sites [23]. At the same time, unstable catechol groups in the molecular structure of DA can coordinate some insufficiently coordinated surface sites, such as Ti atoms, and form charge-transfer complexes between ligands and metals after polymerization into polydopamine [24]. Due to the fact that Ti_4_O_7_ possesses a large amount of unsaturated titanium, DA is used to modify Ti_4_O_7_ powders to improve the dispersibility of the Ti_4_O_7_ in the polyaniline coating and then improve the protection performance of the coating. 

In the present study, the polyaniline coating on 316L stainless steel was modified by the PDA-functionalized Ti_4_O_7_ particles, and the corrosion behaviors of the uncoated and coated 316L in a simulated PEMFC environment were also investigated.

## 2. Experimental Procedures

### 2.1. Materials and Reagents 

The 316L stainless steel with a size of 10 mm × 15 mm × 2 mm was used as the substrate, which was pretreated by sequential grinding with 240# and 600# grit emery paper, cleaned with distilled water and acetone, and then dried. The Ti_4_O_7_ powders were supplied by Songshan Lake Materials Laboratory (Dongguan, China) (99.99%). The used aniline (An, 99%), sulfuric acid (99%), dopamine hydrochloride (DA, 98%), and trimethyl aminomethane (Tris, ≧99.9%) were purchased from Shanghai Maclin Biochemical Technology Co., Ltd (Shanghai, China). They were all analytical-reagent-grade and used without any purification. 

### 2.2. Preparation of PDA-Ti_4_O_7_ Composite Powders

Pristine Ti_4_O_7_ powders were easy to agglomerate, which affected the dispersion effect. Thus, the Ti_4_O_7_ powders were modified by DA to obtain a homogenous dispersion in polyaniline coating. The modification process was as follows: 0.45 g Ti_4_O_7_ was first added to 450 mL Tris buffer solution (0.01 mol/L, pH = 8.5) under continuous ultrasonic treatment for 45 min, and then 0.5 g DA was added to the above mixtures under continuous ultrasonic treatment for 24 h at 30 °C. The mixed solution was finally centrifuged at 8000 rpm for 5 min, and the precipitates were repeatedly washed with deionized water to remove the impurities and further dried at 65 °C for 12 h under vacuum to obtain PDA-Ti_4_O_7_ composite powders.

### 2.3. Electrodeposition of PANI and PANI/PDA-Ti_4_O_7_ Coatings

The electrochemical synthesis of PANI and PANI/PDA-Ti_4_O_7_ coatings was conducted by constant current method on Gamry Interface 1010 electrochemical workstation with a two-electrode system in which 316L was used as the working electrode and Pt plate was used as the counter electrode. For the electropolymerization of the PANI coating, a constant current density of 2.8 mA/cm^2^ was applied on 316L in 0.3 M aniline + 1 M sulfuric acid for 15 min at 5 °C, while, for the PANI/PDA-Ti_4_O_7_ composite coating, 1.5 g/L PDA-Ti_4_O_7_ powders were added into 0.3 M aniline +1 M sulfuric acid solution. A constant current density of 2.8 mA/cm^2^ was applied on 316L in 0.3 M aniline + 1 M sulfuric acid for 15 min at 5 °C with constant stirring.

### 2.4. Characterization

In order to characterize the corrosion resistance of the coating in the PEMFC environment, accelerated corrosion experiment was used in this paper to measure the electrochemical corrosion behavior of the coating in 1 M H_2_SO_4_ + 2 ppm HF, mainly including potentiodynamic polarization and electrochemical impedance spectroscopy (EIS) measurements. For the potentiodynamic polarization measurement, the scan rate is kept at 20 mV·min^−1^. For the EIS measurement, the frequency range is between 0.01 Hz and 100 kHz, with the amplitude of input sine wave voltage of 10 mV. 

Furthermore, scanning electron microscopy (SEM) and X-ray diffraction (XRD) were used to characterize the powders and coatings.

## 3. Results and Discussion

### 3.1. Characterization of Coatings

**Figure 1:**
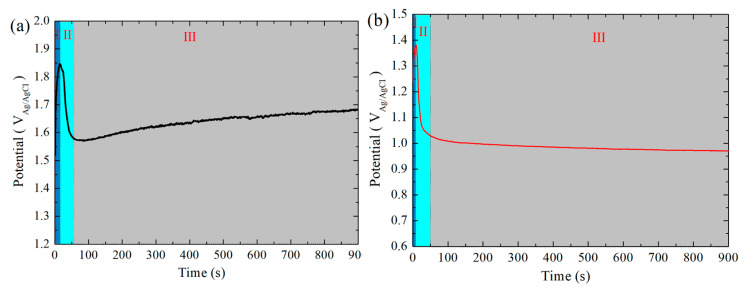
The electropolymerization curves of PANI coating (**a**) and PANI/PDA-Ti_4_O_7_ composite coating (**b**). Dark blue area: I, sky blue area: II, gray area: III.

Figure 1 provides the electropolymerization curves of the PANI coating and the PANI/PDA-Ti_4_O_7_ composite coating. The change in potential can be divided into three stages: I: potential sharply increasing stage. This indicates the nucleation overpotential of aniline on the surface of stainless steel; II: potential slowly declining stage. It is mainly because the formation of polyaniline changes the surface state of 316L; III: potential stabilization stage. It indicates the continuous thickening of the polyaniline coating. For pure polyaniline coating, the nucleation overpotential is about 1.85 V_Ag/AgCl_, and the stabilization potential is about 1.68 V_Ag/AgCl_, which are higher than those of the PANI/PDA-Ti_4_O_7_ composite coating with a nucleation overpotential of about 1.38 V_Ag/AgCl_ and a stabilization potential of about 0.96 V_Ag/AgCl_. This shows that the addition of modified PDA-Ti_4_O_7_ powders reduces the nucleation overpotential and stable synthesis potential, which may be due to the modified powders’ ability to enter the polymer chain through chemical bonding, which is further analyzed in Section 3.5. The relationship between the resultant energy (*Q*) and the coating thickness (*d*) is as follows:

*d* = *QM*/2*Fρ* (1)

in which *M* is the molar molecular weight of aniline, *F* is the Faraday constant, and *ρ* is the density of aniline. Based on the above equation, the thickness of the coating is about 12.6 μm.

**Figure 2:**
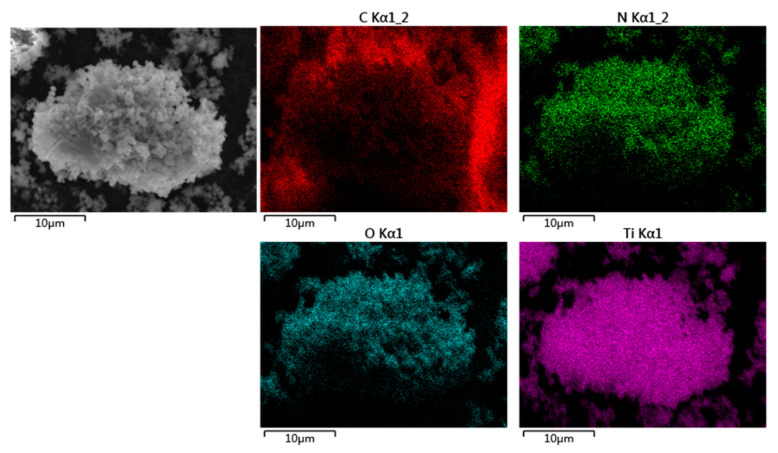
The surface morphology of PDA-Ti_4_O_7_ powders and the corresponding EDS mapping scanning results.

By analyzing the surface morphology of the modified PDA-Ti_4_O_7_ composite powders in Figure 2, it is found that the PDA-Ti_4_O_7_ powders are in the shape of clusters. Combined with the EDS mapping results, it can be seen that Ti belonging to Ti_4_O_7_ and N belonging to the amino group in the molecular structure of PDA are uniformly distributed in the powders, which may be due to the coordination of the catechol groups in PDA with Ti_4_O_7_. This also confirms the successful modification of Ti_4_O_7_ by PDA. 

**Figure 3:**
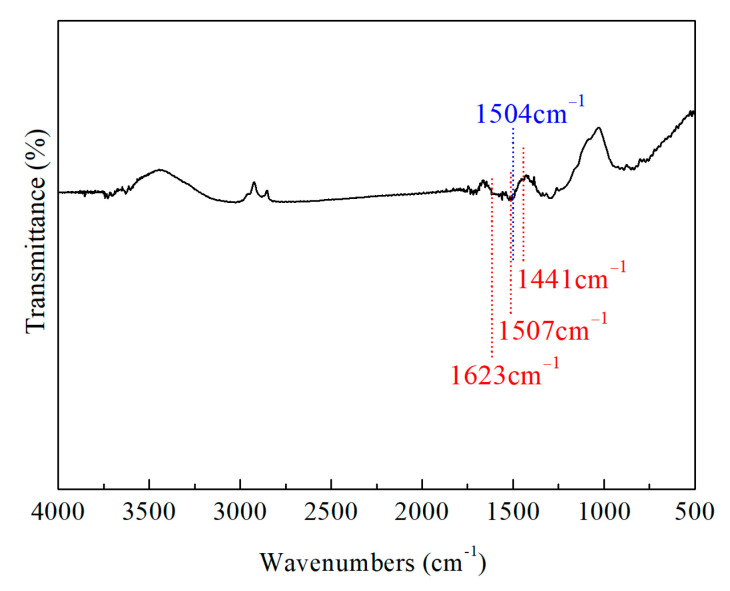
The FT-IR spectra of PDA-Ti_4_O_7_ powders.

To further confirm the chemical bonding between PDA and Ti_4_O_7_, the FT-IR spectra of the PDA-Ti_4_O_7_ powders are provided in Figure 3. The characteristic peak at 1504 cm^−1^ corresponds to -NH bending vibration. In addition, characteristic peaks at 1623 cm^−1^, 1507 cm^−1^, and 1441 cm^−1^ are detected, which correspond, respectively, to the stretching vibration of the benzene ring skeleton. All the above results prove that Ti_4_O_7_ is successfully modified by PDA through the coordination of the stable catechol group with the unsaturated Ti atom in Ti_4_O_7_.

**Figure 4:**
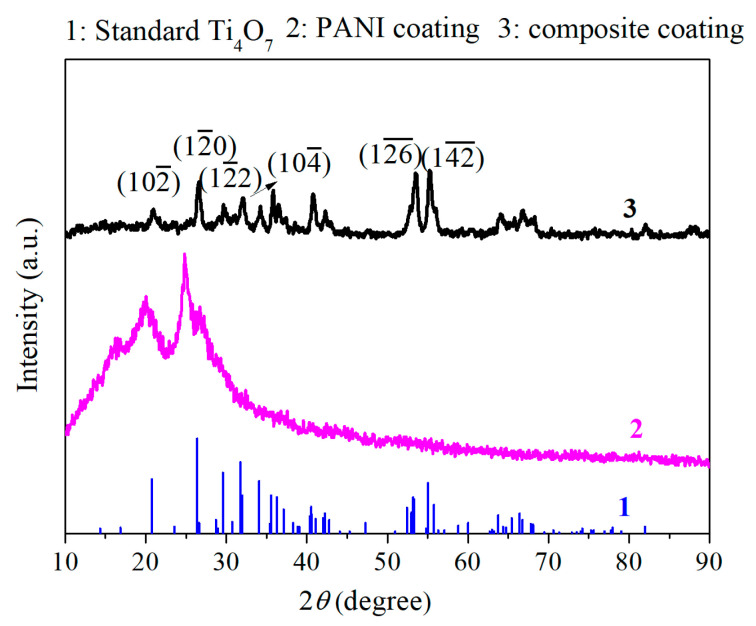
XRD patterns of PANI coating and PANI/PDA-Ti_4_O_7_ composite coating.

Figure 4 provides the XRD patterns of the PANI coating and PANI/PDA-Ti_4_O_7_ composite coating. The PANI coating shows a wide amorphous peak, with two diffraction peaks at 2*θ* about 19.8° and 24.8°, indicating that the PANI coating is mainly amorphous and has local crystallization. The degree of crystallinity of the PANI coating is calculated to be about 9.77% by Jade 6.0, which is similar to the previous reports [13]. The XRD pattern of the PANI/PDA-Ti_4_O_7_ composite coating shows that the diffraction peaks at 2*θ* = 20.7°, 26.3°, 29.5°, and 31.7° are consistent with the standard characteristic diffraction peak diagram for Ti_4_O_7_ [25], which corresponds to crystal planes (1 0 $\bar{2}$), (1 $\bar{2}$ 0), (1 $\bar{2}$ 2), and (1 0 $\bar{4}$), respectively. This means that the PDA-Ti_4_O_7_ powders are successfully doped into the PANI coating.

**Figure 5:**
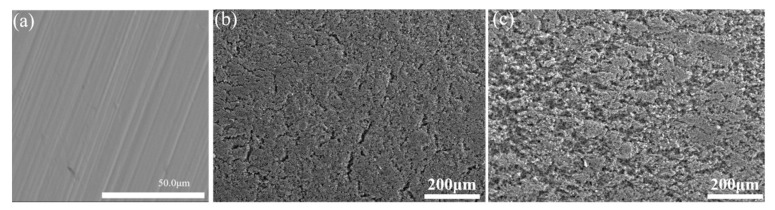
The surface morphologies of 316L (**a**), PANI coating (**b**), and PANI/PDA-Ti_4_O_7_ composite coating (**c**).

**Figure 6:**
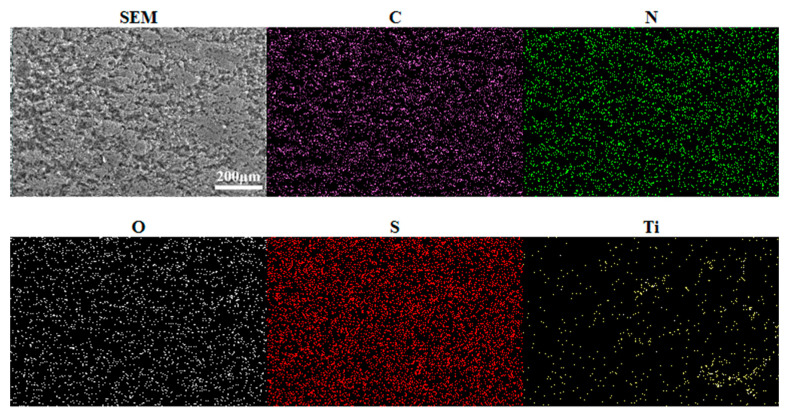
EDS mapping scanning results for the PANI/PDA-Ti_4_O_7_ composite coating.

From the surface morphologies of the 316L substrate, PANI coating, and PANI/PDA-Ti_4_O_7_ composite coating, as shown in Figure 5, the scratches produced during the grinding process are clearly visible on the 316L substrate, which is beneficial to the binding force of the coating. The PANI coating is relatively flat but with some cracks, while the PANI/PDA-Ti_4_O_7_ composite coating is coarse and compact. At the same time, the distribution of Ti in the coating is relatively uniform (Figure 6), which also indicates that the PDA-Ti_4_O_7_ powders are evenly dispersed in the coating due to the chemical bonding between PDA-Ti_4_O_7_ and PANI.

### 3.2. Potentiodynamic Polarization Curves

**Figure 7:**
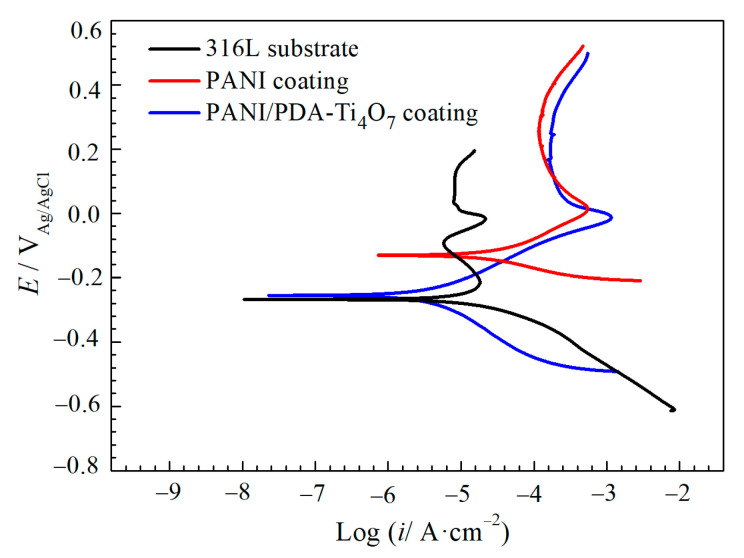
Potentiodynamic polarization curves for 316L, PANI coating, and PANI/PDA-Ti_4_O_7_ composite coating in 1 M H_2_SO_4_ + 2 ppm HF.

Figure 7 shows the potentiodynamic polarization curves for 316L, the PANI coating, and the PANI/PDA-Ti_4_O_7_ composite coating in 1 M H_2_SO_4_ + 2 ppm HF. It can be seen that 316L exhibits typical passivation–activation behavior in the anode region, while the PANI coating and PANI/PDA-Ti_4_O_7_ composite coating show active dissolution behavior in the anode region. Since the anodic Tafel linear region of the polarization curve of 316L is not obvious, a single cathodic Tafel fitting method is used to fit the polarization curve of 316L. Table 1 shows the corrosion potential (*E*_corr_) and corrosion current density (*i*_corr_) of the different samples. The *E*_corr_ of 316L, the PANI coating, and the PANI/PDA-Ti_4_O_7_ composite coating are −265, −130, and −255 mV_Ag/AgCl_, respectively. The *i*_corr_ of 316L is 23.4 μA·cm^−2^, while that of the PANI coating increases to 35.7 μA·cm^−2^, which is due to the special phenomenon caused by the good oxidation deoxidizing ability of PANI itself. The *i*_corr_ of the PANI/PDA-Ti_4_O_7_ composite coating is the smallest, with a value of about 4.05 μA·cm^−2^.

### 3.3. Open-Circuit Potential Curves

**Figure 8:**
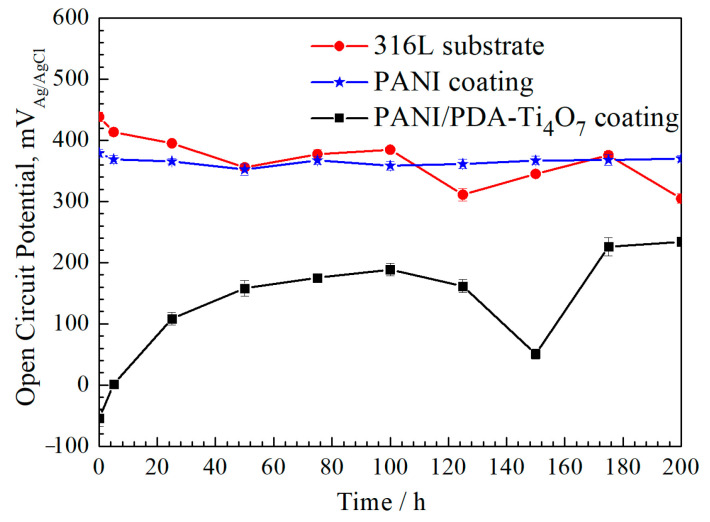
Open-circuit potential curves for 316L, PANI coating, and PANI/PDA-Ti_4_O_7_ composite coating in 1 M H_2_SO_4_ + 2 ppm HF.

The change curves of open-circuit potential for the 316L, 316L/PANI, and 316L/PANI/PDA-Ti_4_O_7_ samples during immersion in 1 M H_2_SO_4_ + 2 ppm HF for 200h are provided in Figure 8. The open-circuit potential of 316L rises slowly during the soaking process, which corresponds to the formation process of the surface passivation film. Then, it decreases significantly after immersion for 125 h (about 46 mV_Ag/AgCl_), which corresponds to the process of the destruction of the passivation film and the further corrosion process. The open-circuit potential of the 316L/PANI sample shows a trend of fluctuation decline and drops to about 304 mV_Ag/AgCl_ after soaking for 200 h, which is related to the corrosion of the substrate caused by corrosive ions penetrating the coating. The open-circuit potential of the 316L/PANI/PDA-Ti_4_O_7_ sample remains stable with a value of about 365 mV_Ag/AgCl_ in the prolonged soaking time. The open-circuit potential of the composite coating is higher and more stable than that of the single PANI coating and bare 316L, indicating that the composite coating has a continuous and efficient protection effect on the 316L substrate.

### 3.4. Electrochemical Impedance Spectra Curves

**Figure 9:**
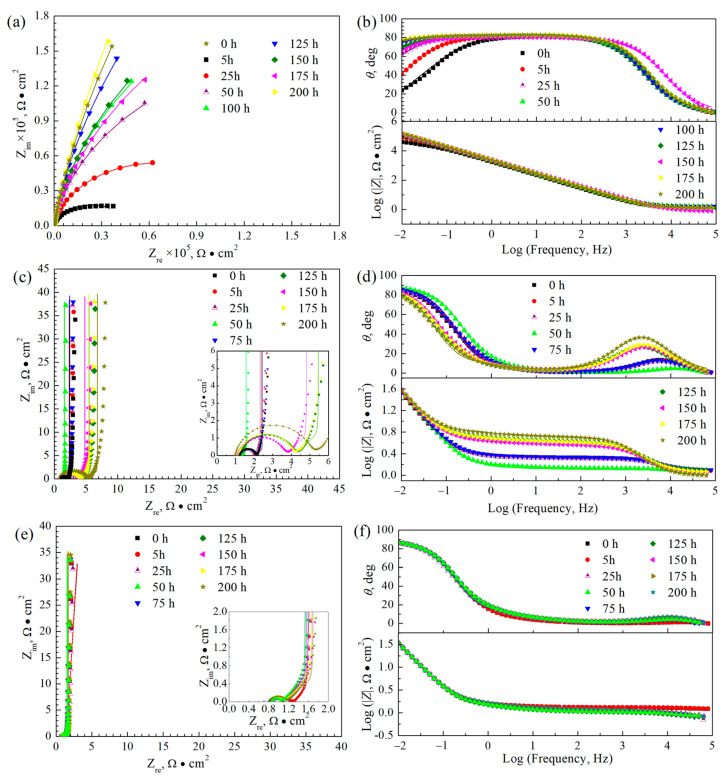
Nyquist and Bode plots for 316L (**a**,**b**), PANI coating (**c**,**d**), and PANI/PDA-Ti_4_O_7_ composite coating (**e**,**f**) in 1 M H_2_SO_4_ + 2 ppm HF at 25 °C, respectively. Point: experimental data; line: simulated date.

**Figure 10:**
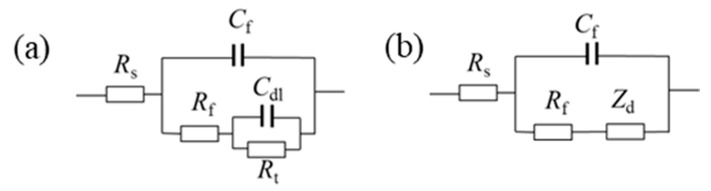
Equivalent circuit for fitting the impedance spectra of 316L (**a**) and PANI coating and PANI/PDA-Ti_4_O_7_ composite coating (**b**).

Figure 9 presents the Nyquist and Bode plots for 316L, the PANI coating, and the PANI/PDA-Ti_4_O_7_ composite coating in 1 M H_2_SO_4_ + 2 ppm HF at 25 °C, respectively. According to the change characteristics of the curve in the Nyquist diagrams in Figure 9, the Nyquist curve can be divided into a high-frequency region and low-frequency region, in which the change in the Nyquist curve in the high-frequency region mainly reflects the electrochemical behavior of the coating or oxide layer while the change in the Nyquist curve in the low-frequency region represents the electrochemical response information at the matrix/coating interface. For the 316L sample, the Bode plots clearly show two time constants, while only one capacitive loop can be observed in the Nyquist plots. Therefore, an equivalent circuit in Figure 10a is chosen for fitting the impedance spectra of 316L, in which *R*_s_ is the solution resistance, *R*_t_ and *C*_dl_ represent the double-layer resistance and double-layer capacitance, respectively, and *R*_f_ and *C*_f_ represent the resistance and capacitance of the corrosion layer. Considering the dispersion effect, the pure capacitor *C* is replaced by a constant phase angle element *Q* when fitting and can be expressed as *Z*_CPE_ = *Y*_0_^−1^(*jw*)^−n^. The fitting results are represented in Figure 9a,b and Table 2. It can be seen that *R*_t_ and *R*_f_ increase in the initial 10 h, indicating the formation of an oxide layer, and the corrosion begins. However, *R*_f_ sharply decreases during the 25~125 h immersion, which may be due to the destruction of the passivation film. Due to the re-passivation behavior of 316L, *R*_f_ increases again in the later immersion stage.

**Figure 11:**
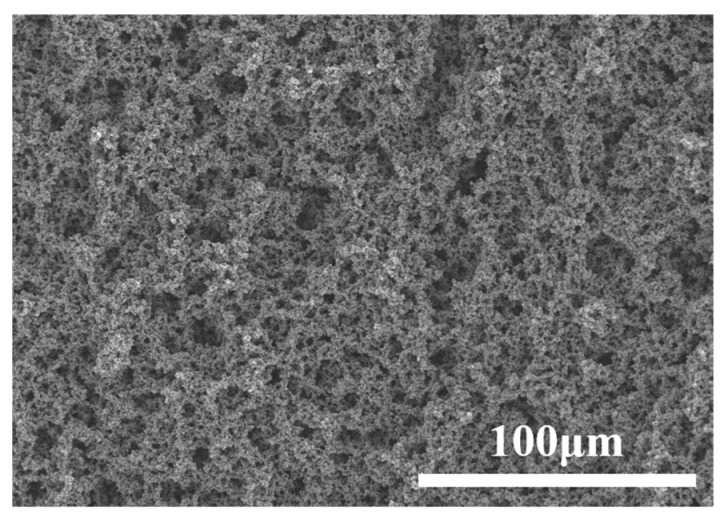
The surface morphology of PANI/PDA-Ti_4_O_7_ composite coating after 200 h immersion in 1 M H_2_SO_4_ + 2 ppm HF.

For the PANI coating and PANI/PDA-Ti_4_O_7_ composite coating samples, the Nyquist plots clearly consist of a capacitive arc at a high frequency and a straight line at a low frequency. The low-frequency region shows the characteristic of effective barrier layer diffusion; i.e., the straight line is almost perpendicular to the real axis. At the same time, the Bode plots clearly show two time constants. Based on the above characteristics, an equivalent circuit in Figure 10b is chosen for fitting the impedance spectra of the PANI coating and PANI/PDA-Ti_4_O_7_ composite coating. In Figure 10b, *Z*_d_ represents the impedance of the effective diffusion layer and can be expressed as *Z*_d_ = *T*(*jw*)^−1/2^ Coth[*t*(*jw*)^1/2^]. The fitting results are represented in Figure 9c–f and Table 3 and Table 4. It can be seen that *R*_f_ of both the PANI coating and PANI/PDA-Ti_4_O_7_ composite coating decreases in the initial immersion stage, which can be ascribed to the increased conductivity caused by the infiltration of corrosive ions through the micropores in the coating. When the immersion time is increased to 175 h, the *R*_f_ of the PANI coating sharply increases, indicating that the coating cannot remain in a protective state. Meanwhile, the *R*_f_ of the PANI/PDA-Ti_4_O_7_ composite coating shows no significant increase during the 200 h immersion, indicating that the composite coating can provide effective protection for the substrate. This can be explained by the oxidation–reduction state of PANI. For the PANI coating, the PANI coating can make the 316L substrate passivated with the infiltration of the corrosive ions. At the same time, the PANI will be reduced, resulting in a decrease in conductivity. In addition, the PANI will be secondarily doped by SO_4_^2−^ and oxygen in the corrosion solution; therefore, the corrosion potential fluctuates during immersion, as shown in Figure 8. With the addition of conductive PDA-Ti_4_O_7_ powders, the high corrosion potential makes the secondary doping process of PANI by SO_4_^2−^ and oxygen in the corrosion solution much faster than that for the pure PANI coating, thus increasing the conductivity. The slight increase in *R*_f_ may be related to the re-passivation behavior of 316L. At the same time, the *R*_f_ of the PANI/PDA-Ti_4_O_7_ composite coating is smaller than that of the PANI coating, indicating that the composite coating shows better conductivity. After 200 h of corrosion, the PANI/PDA-Ti_4_O_7_ composite coating can still maintain a relatively compact morphology (Figure 11).

### 3.5. Corrosion Mechanism

**Figure 12:**
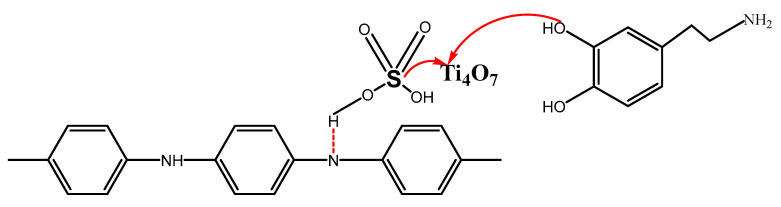
Formation mechanism of PANI/PDA-Ti_4_O_7_ composite coating.

Based on the above results, compared with the PANI coating, the PANI/PDA-Ti_4_O_7_ composite coating shows higher corrosion resistance. This is mainly due to the addition of PDA-Ti_4_O_7_ particles enhancing the effect of the physical barrier layer and reducing the porosity of the PANI coating. H in sulfuric acid forms a hydrogen bond with polyaniline through electrostatic interaction to form doped polyaniline. Ti is unsaturated and coordinated with O in dopamine to generate modified Ti_4_O_7_. Ti can also coordinate with S in doped polyaniline to connect PANI, Ti_4_O_7_, and PDA, which has been proved by the work in Ref. [25], as shown in Figure 12. Thus, the formation of a chemical bond between PANI, Ti_4_O_7_, and PDA makes the powder evenly dispersed in the PANI coating. Furthermore, Ti_4_O_7_ shows high chemical stability, with a higher oxidation–reduction potential, which can cause PANI to be in an oxidation state. Therefore, the PANI/PDA-Ti_4_O_7_ composite coating can provide effective anodic protection for 316L. 

## 4. Conclusions

By combining the organic polymer PANI with the modified conductive particle PDA-Ti_4_O_7_, a PANI/PDA-Ti_4_O_7_ composite coating with better performance was obtained. PDA-Ti_4_O_7_ particles were doped in the PANI coating successfully, and the distribution was uniform. In the electrochemical corrosion test in 1 M H_2_SO_4_ + 2 ppm HF, the composite coating maintains the highest and stable open-circuit potential (365 mV_Ag/AgCl_). The corrosion current density of the composite coating is 4.05 μA·cm^−2^, and the potentiodynamic polarization data show that the composite coating has good corrosion resistance. The relevant data of electrochemical impedance spectroscopy show that the composite coating can maintain long-term corrosion resistance in the internal working environment of PEMFCs.

## Figures and Tables

**Table 1:** Electrochemical parameters of 316L, PANI coating, and PANI/PDA-Ti_4_O_7_ composite coating were obtained using the Tafel extrapolation method.

Samples	*E*_corr_ (mV)	*i*_corr_ (μA·cm^−2^)
316L	−265	23.4
PANI coating	−130	35.7
PANI/PDA-Ti_4_O_7_ composite coating	−255	4.05

**Table 2:** Fitting data for the impedance spectrum of 316L in 1 M H_2_SO_4_ + 2 ppm HF.

Time (h)	*R*_s_ (Ω·cm^2^)	*Y*_f_ (Ω^−1^·cm^−2^·S^−n^)	*n*_f_	*R*_f_ (Ω·cm^2^)	*Y*_dl_ (Ω^−1^·cm^−2^·S^−n^)	*n*_dl_	*R*_t_ (Ω·cm^2^)
0	1.38	1.03 × 10^−4^	0.91	3.35 × 10^4^	5.87 × 10^−4^	0.99	1.10 × 10^4^
5	1.39	9.23 × 10^−5^	0.91	9.89 × 10^4^	9.62 × 10^−5^	0.60	3.78 × 10^4^
25	1.43	7.52 × 10^−5^	0.92	14.82	1.45 × 10^−5^	0.81	3.49 × 10^5^
50	1.56	7.73 × 10^−5^	0.92	15.22	1.02 × 10^−5^	0.83	5.60 × 10^5^
100	1.57	6.91 × 10^−5^	0.93	8.66	1.26 × 10^−5^	0.84	1.13 × 10^6^
125	1.30	7.34 × 10^−5^	0.92	7.87	1.47 × 10^−5^	0.83	6.26 × 10^5^
150	0.80	6.79 × 10^−5^	0.91	0.34 × 10^3^	1.18 × 10^−5^	0.79	5.55 × 10^5^
175	1.37	7.37 × 10^−5^	0.92	1.42 × 10^5^	2.75 × 10^−6^	0.66	2.56 × 10^6^
200	1.36	7.50 × 10^−5^	0.92	1.04 × 10^5^	2.90 × 10^−6^	0.66	1.75 × 10^6^

**Table 3:** Fitting data for the impedance spectrum of PANI coating in 1 M H_2_SO_4_ + 2 ppm HF.

Time (h)	*R* (Ω·cm^2^)	*Y*_f_ (Ω^−1^·cm^−2^·s^n^)	*n*_f_	*R*_f_ (Ω·cm^2^)	*T* (Ω^−1^·cm^−2^·s^0.5^)	*t* (s^0.5^)
0	1.22	5.79 × 10^−5^	0.97	0.84	0.64	0.70
5	1.24	6.54 × 10^−5^	0.96	0.78	0.64	0.67
25	1.24	6.24 × 10^−5^	0.97	0.74	0.62	0.66
50	1.11	6.27 × 10^−5^	0.99	0.23	0.76	0.55
75	1.26	7.43×10^−5^	0.95	0.81	0.60	0.67
125	1.24	1.25 × 10^−4^	0.89	2.85	0.32	1.32
150	1.13	1.16 × 10^−4^	0.90	2.47	0.33	1.22
175	1.16	1.08 × 10^−4^	0.90	2.90	0.32	1.28
200	0.98	1.00 × 10^−4^	0.90	4.05	0.27	1.46

**Table 4:** Fitting data for the impedance spectrum of PANI/PDA-Ti_4_O_7_ composite coating in 1 M H_2_SO_4_ + 2 ppm HF.

Time (h)	*R* (Ω·cm^2^)	*Y*_f_ (Ω^−1^·cm^−2^·s^n^)	*n*_f_	*R*_f_ (Ω·cm^2^)	*T* (Ω^−1^·cm^−2^·s^0.5^)	*t* (s^0.5^)
25	0.87	1.03 × 10^−4^	0.94	0.26	0.54	0.90
50	0.88	8.98 × 10^−5^	0.99	0.15	0.55	0.85
75	0.89	7.50 × 10^−5^	0.99	0.17	0.55	0.84
100	0.82	9.00 × 10^−5^	0.99	0.17	0.55	0.84
125	0.83	8.52 × 10^−5^	0.98	0.20	0.55	0.84
150	0.85	9.79 × 10^−5^	0.97	0.22	0.54	0.84
175	0.86	1.06 × 10^−4^	0.97	0.24	0.53	0.86
200	0.85	1.17 × 10^−4^	0.95	0.28	0.51	0.88

## Data Availability

The original contributions presented in the study are included in the article, further inquiries can be directed to the corresponding author.
